# SMA-YOLO: A Novel Approach to Real-Time Vehicle Detection on Edge Devices

**DOI:** 10.3390/s25165072

**Published:** 2025-08-15

**Authors:** Haixia Liu, Yingkun Song, Yongxing Lin, Zhixin Tie

**Affiliations:** 1 Keyi College, Zhejiang Sci-Tech University, Shaoxing 312369, China; 2 School of Computer Science and Technology, Zhejiang Sci-Tech University, Hangzhou 310018, China

**Keywords:** vehicle detection, YOLOv7, light weighting, SimAM attention mechanism, MobileNetV3, ACON activation function

## Abstract

Vehicle detection plays a pivotal role in traffic management as a key technology for intelligent traffic management and driverless driving. However, current deep learning-based vehicle detection models face several challenges in practical applications. These include slow detection speeds, large computational and parametric quantities, high leakage and misdetection rates in target-intensive environments, and difficulties in deploying them on edge devices with limited computing power and memory. To address these issues, this paper proposes an improved vehicle detection method called SMA-YOLO, based on the YOLOv7 model. Firstly, MobileNetV3 is adopted as the new backbone network to lighten the model. Secondly, the SimAM attention mechanism is incorporated to suppress background interference and enhance small-target detection capability. Additionally, the ACON activation function is substituted for the original SiLU activation function in the YOLOv7 model to improve detection accuracy. Lastly, SIoU is used to replace CIoU to optimize the loss of function and accelerate model convergence. Experiments on the UA-DETRAC dataset demonstrate that the proposed SMA-YOLO model achieves a lightweight effect, significantly reducing model size, computational requirements, and the number of parameters. It not only greatly improves detection speed but also maintains higher detection accuracy. This provides a feasible solution for deploying a vehicle detection model on embedded devices for real-time detection.

## 1. Introduction

Intelligent traffic systems [1,2] play a crucial role in urban transportation planning, traffic accident reduction, and vehicle–road coordination. Vehicle detection technology, as the core and foundation of intelligent traffic systems, has gained significant attention for its applications in unmanned driving [3,4], traffic accident detection [5,6], driving violation detection [7,8], and vehicle path planning [9]. However, current vehicle detection technology still has problems with limited detection accuracy and speed, which cannot meet the requirements for real-time and accurate vehicle detection. Additionally, the existing vehicle detection algorithms are complex and require high computational capabilities, making them difficult to deploy on embedded devices.

Recent advancements in vehicle detection have evolved from traditional computer vision techniques to deep learning-based approaches. Early methods relied on handcrafted features such as Haar cascades [10] and the Histogram of Oriented Gradient-Support Vector Machine (HOG-SVM) [11], which struggled with occlusions and lighting variations. The emergence of convolutional neural networks significantly improved robustness, with two-stage detectors like Faster Region-based Convolutional Neural Network (Faster R-CNN) [12] achieving high accuracy at the cost of real-time performance. Subsequent one-stage detectors, including You Only Look Once (YOLO) [13] and its variants (e.g., YOLOv4 [14], YOLOv7 [15]), prioritized speed by simplifying network architectures. Despite these advancements, there is still a need for further improvements in detection accuracy and speed to fully meet the demands of real-time and precise vehicle detection in intelligent traffic systems.

To address the problems of complex structures, large numbers of parameters and computational costs in existing vehicle detection models, as well as the difficulty in deploying them on embedded devices with limited storage and computation capacity, in this paper, we modify the backbone model of YOLOv7 [15] to MobileNetV3 [16], constructing a lightweight vehicle detection model. To compensate for the loss of accuracy caused by the light-weighted backbone network, we firstly replace the initial Sigmoid Linear Unit (SiLU) activation function in the YOLOv7 network with the Adaptive Conjugate One-Nonlinear Activation (ACON) function [17], which improves the model’s generalization ability and the efficiency of the information transfer in each feature layer. Next, we incorporate the Simple Parameter-Free Attention Module (SimAM) [18] into the backbone network, which improves the model’s ability to extract features. Finally, we use SCYLLA Intersection over Union (SIoU) [19] to optimize the loss function, which improves the model’s detection performance. Experimental results demonstrate that the proposed SMA-YOLO model not only improves the detection speed and meets the real-time requirements for vehicle detection but also enhances accuracy and detection performance.

The main contributions of this paper are as follows:

(1) A lightweight road vehicle detection model called SMA-YOLO for embedded devices with limited storage and computing power is constructed by replacing the backbone network of the YOLOv7 model with MobileNetV3.
(2) The ACON activation function is introduced to replace the SiLU activation function in the original YOLOv7 model, which enhances the model’s generalization ability and the information transfer efficiency between feature layers.
(3) The SimAM attention mechanism module is integrated in the backbone network, which effectively improves the feature extraction ability of the model.
(4) The SIoU is adopted as the loss function to optimize the model and further enhance the detection performance.

## 2. Related Work

Current vehicle detection techniques can be categorized into three main approaches: traditional vision-based detection, wireless sensor-based and deep learning-based target detection algorithms. Vision-based techniques typically follow three stages: selecting the target region, extracting target feature information, and classifying these features. However, these methods suffer from several drawbacks: redundant windows, being time-consuming, low detection efficiency, high rates of missed and false detections, stringent equipment requirements, and the poor robustness and generalization performance of manually designed features. These limitations make it difficult to complete detection tasks in complex environments. Sensor-based vehicle detection methods, which use ultrasonic [20,21], microwave [22,23], geomagnetic [24,25] or inductive coil technology [26,27], represent the current mainstream approach for parking lot vehicle detection. The disadvantages of these methods include a cumbersome installation process, strict environmental requirements, and inapplicability to complex road traffic scenes. With the development of deep-learning technology, target detection methods based on deep learning have gradually become a prominent research topic [28,29,30,31,32]. The current target detection algorithms based on deep learning can be divided into two categories: single-stage regression-based algorithms and two-stage region proposal-based algorithms. The representative two-stage algorithms include Region-based Convolutional Neural Network (R-CNN) [33], Fast R-CNN [34], Faster R-CNN [12], etc. These algorithms offer higher localization precision and accuracy but suffer from slower training speeds and lower detection rates. Conversely, single-stage detection algorithms, such as Single Shot Multi-Box Detector (SSD) [35], YOLO [13], EfficientDet [36], and RetinaNet [37], provide high real-time performance and fast detection rates, at the cost of slightly lower accuracy compared to two-stage algorithms.

In recent years, many scholars have proposed a series of improvements to address the shortcomings of different target detection algorithms. Li et al. [38] proposed the Cascade Multi-Scale Region-based Convolutional Neural Network (CMS R-CNN) algorithm which utilizes cascaded multi-scale regions to combine high-resolution information with rich semantic information, thus alleviating the difficulty in detecting small vehicles. Li et al. [39] introduced a cross-layer fusion multi-object detection and recognition algorithm based on Faster R-CNN. This approach uses the five-layer structure of VGG16 (Visual Geometry Group) to obtain more characteristic information, achieving better effects. Chen et al. [40] incorporated the Inception module into the SSD algorithm, improving the detection accuracy while maintaining a higher detection rate. Simon et al. [41] used the sum of imaginary and real fractions and a specific regression strategy to improve the YOLOv2 model to obtain the Euler-Region-Proposal Network (E-RPN) model, which further improves detection accuracy.

In the study of vehicle detection algorithms for embedded systems and resource-constrained devices, researchers have developed numerous efficient and lightweight vehicle detection networks for these platforms. Ge et al. [42] proposed a lightweight vehicle detection network based on an improved YOLOv3-tiny model. They optimized the feature extraction network by replacing the original feature extractor with DarkNet-19 and ResNet-18, enhancing detection accuracy. Furthermore, they used the K-means algorithm to cluster nine anchor boxes for multi-scale prediction, especially for small targets. Wu et al. [43] enhanced YOLOv3-tiny to develop a lightweight vehicle detection network. They introduced a spatial pyramid pooling structure and a K-mean++ clustering algorithm to enhance feature extraction and bounding box prediction. Moreover, they replaced the Intersection over Union (IoU) loss function with the Generalized Intersection over Union (GIoU) loss to improve localization accuracy. The model was compressed by pruning redundant channels using the scaling factor of the batch normalization layer. Taheri Tajar et al. [44] developed a lightweight YOLOv3-tiny vehicle detection model by pruning and simplifying the original network. They trained the model on the BIT-Vehicle dataset and excluded unnecessary layers. Collectively, these studies demonstrate that lightweight modifications to YOLOv3-tiny can significantly improve detection performance while maintaining real-time speed in embedded systems and resource-constrained devices. Yuan and Xu [45] proposed a lightweight vehicle detection algorithm by improving YOLOv4. They replaced the original CSPDarknet53 backbone network with MobileNetv3 for feature extraction and used deep separable convolution in the enhanced feature extraction networks Spatial Pyramid Pooling (SPP) and Path Aggregation Network (PANet) to further reduce parameters. Additionally, the loss function was redesigned using a weighting method to address the imbalance in object detection data. The optimized YOLOv4 model improved accuracy by 0.53% while reducing model parameters by 78%, showing its effectiveness in achieving a balance between accuracy and efficiency. Bie et al. [46] proposed the YOLOv5n-L algorithm for real-time vehicle detection. They incorporated depth-wise separable convolution and the C3Ghost module to reduce model parameters and improve detection speed. A Squeeze-and-Excitation attention mechanism was integrated into the backbone network to enhance accuracy and suppress environmental interference. Furthermore, a bidirectional feature pyramid network was employed for multi-scale feature fusion. Compared to comparison models, this method has higher detection accuracy and lower computational costs and reduces the platform storage and computing capacity requirements, so can be easily employed in resource-constrained devices. Liu et al. [47] introduced YOLOv8-FDD, employing feature-sharing and dynamic-interaction detection heads alongside multi-scale feature enhancements to achieve a more lightweight and accurate model for real-time vehicle detection in traffic scenarios. Xie et al. [48] presented YOLO-ACE, optimizing the YOLOv10 backbone and neck architecture and applying knowledge distillation to create a highly efficient detector with improved accuracy for vehicle and pedestrian perception in autonomous driving.

YOLOv7 has demonstrated superior performance in vehicle detection tasks across diverse scenarios, achieving an optimal balance between speed and accuracy. Its architecture, featuring Extended Efficient Layer Aggregation Network (E-ELAN) modules and compound model scaling, optimizes gradient paths and computational efficiency, enabling robust multi-scale feature extraction critical for detecting vehicles of varying sizes in complex urban or aerial environments [15,49]. YOLOv7-RAR [50] enhances YOLOv7 with a Res3Unit backbone and ACmix hybrid attention to improve small-target recognition under occlusions and lighting variations. Lightweight adaptations of YOLOv7 further extend its applicability. For instance, YOLOv7-RDD [51] employs DSConv and SimAM attention to reduce computational complexity while maintaining high precision in pavement distress detection. Though focused on road defects, these methodologies—including spatial pyramid pooling optimizations (SPPCSPD) and SIoU loss—are transferable to vehicle detection, particularly enhancing small-object localization and reducing redundant predictions.

## 3. Improved Model SMA-YOLO Based on YOLOv7

The proposed model, SMA-YOLO, is an improved YOLOv7-based vehicle detection method featuring three key upgrades: (1) replacing the backbone with MobileNetV3 to reduce parameters; (2) incorporating SimAM attention to enhance small-target feature extraction; and (3) swapping SiLU activations with ACON to boost detection accuracy. Additional optimization includes adopting SIoU loss for faster convergence.

**Figure 1:**
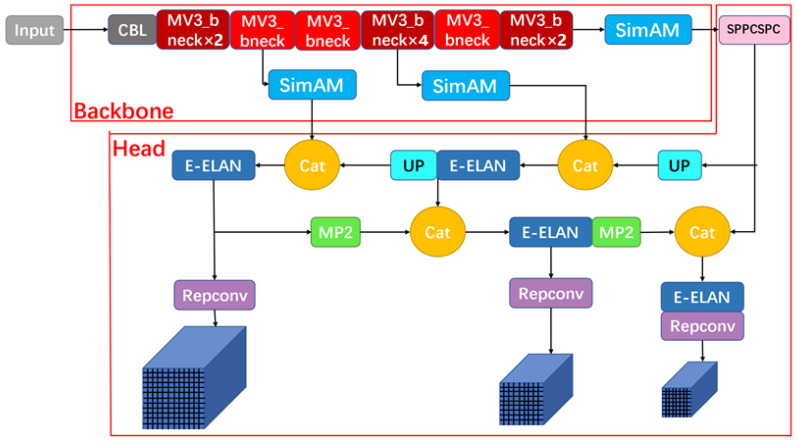
Overall structure of the SMA-YOLO model.

**Figure 2:**
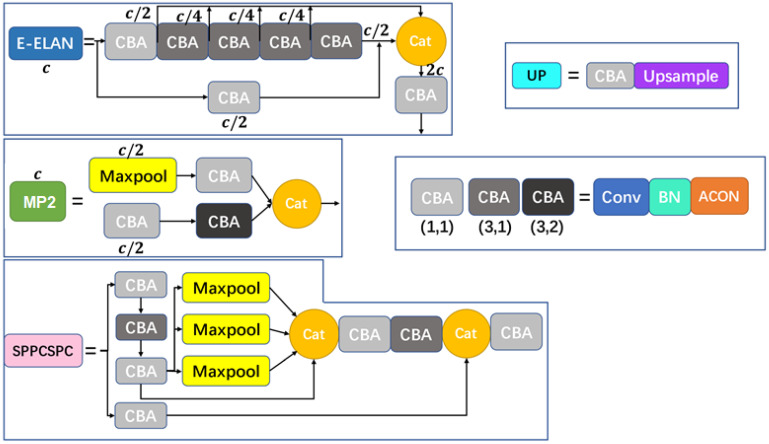
Structure of each module in the SMA-YOLO model.

The SMA-YOLO model structure is shown in Figure 1, and the detailed structure of each module is shown in Figure 2. In particular, the structure of the CBA module is Conv+BN+ACON, which accelerates and optimizes the convolutional neural network. The efficient aggregation network E-ELAN can improve the accuracy and efficiency of target detection. The SPPCSPC structure connects the SPP (Spatial Pyramid Pooling) module and the CSPC (Cross Stage Partial Connections) module in series to obtain richer feature representation, which improves the accuracy of the image classification task while guaranteeing the high efficiency of the network. The main role of the MP2 module is to introduce multiscale information into the deep neural network, so as to improve the network’s target object perception and classification accuracy.

### 3.1. Feature Extraction Network MobileNetV3

To reduce model complexity, this paper introduces MobileNetV3 [16] as a substitute for the original backbone network in YOLOv7. MobileNetV3 is available in two versions: MobileNetV3-Large and MobileNetV3-Small, with differences in the number of channels and the number of the bottlenecks. In this paper, MobileNetV3-Small is adopted; its network structure is detailed in Table 1.

**Figure 3:**
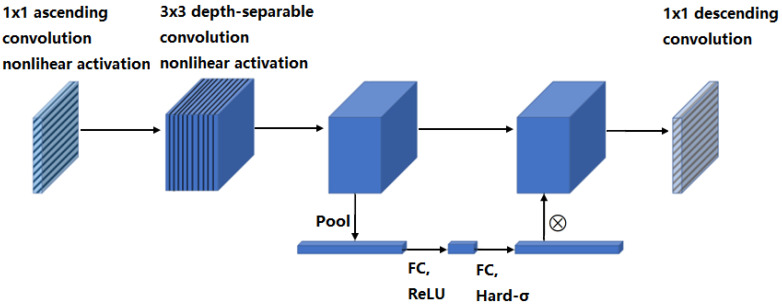
Structure of the bottleneck module in MobileNetV3-Small network.

The structure of the bottleneck module in the network is shown in Figure 3. The feature information extracted from the high-dimensional space was firstly analyzed using a 1 × 1 ascending convolution, followed by a 3 × 3 depth-separable convolution, to which the SE (Squeeze-and-Excitation) attention mechanism was introduced to adjust the weight sizes of the different channels. Finally, a 1 × 1 descending convolution operation was performed, and residual operations were performed on the input and output results when the step size stride = 1.

### 3.2. ACON Activation Function

In this paper, the ACON [17] activation function is adopted to replace the original SiLU function in the YOLOv7 model, as shown in the definition of the CBA module in Figure 2. The ACON activation function was obtained by approximately smoothing the Maxout series function, which can adaptively control the activation function linearly or nonlinearly, and has the advantages of non-saturation and sparsity, which can reduce the negative impact of neuronal necrosis. This specially designed activation function enables each neuron to adaptively choose whether to activate or not, which helps to improve the generalization ability of the model and the information transfer efficiency of each feature layer, while also reducing the computation and complexity of the model to a certain extent.

ACON-C is a generalized activation framework in the ACON family [17], defined using Equation (1):

(1) $$f\left(x\right)=(p_{1}-p_{2})x\cdot \sigma [\beta (p_{1}-p_{2})x)]+p_{2}x$$

where $p_{1}$ and $p_{2}$ are two learnable parameters that functionally control the values of the upper and lower bounds, which can be adaptively tuned in the network; σ denotes the sigmoid activation function; and β is a smoothing factor that controls whether the neuron is activated (β is 0 for no activation).

### 3.3. SimAM Attention Mechanisms

Existing attention modules usually work through a serial or parallel combination of spatial and channel attention mechanisms to facilitate information selection during visual processing. However, in the human brain, these two attentional mechanisms usually work in parallel. Therefore, we introduced the SimAM attention mechanism [18] to assign unique weights to each neuron. The location of the SimAM attention mechanism introduced into SMA-YOLO is shown in Figure 1.

SimAM is a parameter-free attention mechanism based on an energy function, which calculates the importance weight of each neuron directly by optimizing the energy function. The design of SimAM is inspired by the spatial suppression theory in neuroscience, where active neurons inhibit the activity of surrounding neurons, thereby assigning higher priority to the active neurons. Its core weighting process can be expressed as Equation (2):

(2) $$e_{t}^{*}=\frac{4({\hat{\sigma }}^{2}+\lambda )}{(t-\hat{\mu }{)}^{2}+2{\hat{\sigma }}^{2}+2\lambda }$$

where t is the target neuron, $\hat{\mu }$ is the mean of all neurons except t, ${\hat{\sigma }}^{2}$ is the variance of all neurons except t, λ is the regularization coefficient, used to balance the energy function.

The lower the energy, the more the neuron t is distinguished from the surrounding neurons and the higher the importance. According to the definition of the attentional mechanism, the features need to be augmented using Equation (3):

(3) $$\tilde{X}=sigmoid\left(\frac{1}{E}\right)⊙X$$

where sigmoid is a mathematical function capable of mapping real numbers to a range between 0 and 1, E is an energy matrix used to extract key features in an image or perform other image processing tasks, where each element represents the energy value of a pixel point, ⊙ is a per-element multiplication operator, and X is the feature matrix, which represents the features in the input data. The effect of this formula is to weight the original feature matrix X using a sigmoid function and an energy matrix to produce an enhanced feature matrix $\tilde{X}$.

### 3.4. Loss Function

Localization loss in the YOLOv7 network model is calculated using CIoU, which is calculated using Equation (4):

(4) $$L_{CIoU}=1-I_{IoU}+\frac{\rho^{2}(b,b_{gt})}{c^{2}}+\alpha v$$

where $I_{IoU}$ represents the intersection and concurrency ratio between the predicted and real frames, b represents the predicted frame, $b_{gt}$ represents the real frame, $\rho^{2}(b,b_{gt})$ is the Euclidean distance between the centroid of the predicted frame and the real frame, c represents the diagonal length of the smallest external frame of the predicted and real frames, α denotes the equilibrium parameter, and v is used to measure the disparity in the width-to-height ratio of the predicted frame to the real frame. v and α can be calculated using Equation (5) and Equation (6), respectively.

(5) $$v=\frac{4}{\pi^{2}}(arctan\frac{w_{gt}}{h_{gt}}-arctan\frac{w}{h}{)}^{2}$$

(6) $$\alpha =\frac{v}{\left(1-I_{IoU}\right)+v}$$

where $w_{gt}$ and $h_{gt}$ denote the width and height of the real frame, and w and h denote the width and height of the predicted frame, respectively.

**Figure 4:**
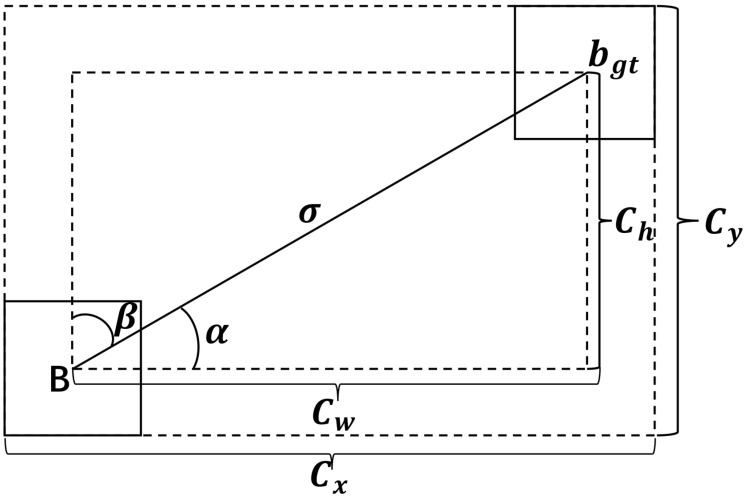
Schematic of the parameters of the SIoU loss function.

When the width-to-height ratio of the predicted frame and the real frame is the same, v is equal to 0. At this time, the effect of the aspect ratio penalty term disappears and the CIoU loss function is less effective. Therefore, the SIoU loss function [19] was selected instead of CIoU in this study. The angular cost is introduced in the SIoU loss function to redescribe the distance, which reduces the total degrees of freedom of the loss function. The meanings of the parameters in the SIoU loss function are shown in Figure 4.

The SIoU loss function is divided into three parts: angle cost, distance cost and shape cost.

(1) Angle Cost

Adding an angle-aware component minimizes the number of distance-related variables. The prediction results were first matched to the X or Y axis, and then the relevant calculations were performed along that axis. α denotes the angular difference between the target frame and the real frame, and the convergence process minimizes α if α≤π/4, otherwise β is minimized. β is computed as shown in Equation (7):

(7) $$\beta =\frac{\pi }{2}-\alpha$$

The angular cost is calculated using Equations (8)–(11):

(8) $$\Lambda =1-2\times {sin}^{2}(\arcsin\left(x\right)-\frac{\pi }{4})$$

(9) $$x=\frac{c_{h}}{\sigma }=sin(\alpha )$$

(10) $$\sigma =\sqrt{(b_{c_{x},gt}-b_{c_{x}}{)}^{2}+(b_{c_{y},gt}-b_{c_{y}}{)}^{2}}$$

(11) $$c_{h}=\max\left(b_{c_{y},gt},b_{c_{y}}\right)-\min\left(b_{c_{y},gt},b_{c_{y}}\right)$$

where $b_{c_{x}}$ and $b_{c_{y}}$ denote the center coordinates of the prediction frame, and $b_{c_{x},gt}$ and $b_{c_{y},gt}$ denote the center coordinates of the real frame.

(2) Distance Cost

Distance cost measures the distance between the center point of the real frame and the predicted frame and is calculated using Equation (12):

(12) $$\Delta =\sum_{t=x,y}\left(1-e^{-\gamma \rho_{t}}\right)$$

where γ and $\rho_{t}$ are hyperparameters used to regulate the distance cost function, and $\rho_{x}$, $\rho_{y}$ and γ are calculated using Equation (13), Equation (14) and Equation (15), respectively:

(13) $$\rho_{x}=(\frac{b_{c_{x},gt}-b_{c_{x}}}{c_{w}}{)}^{2}$$

(14) $$\rho_{y}=(\frac{b_{c_{y},gt}-b_{c_{y}}}{c_{h}}{)}^{2}$$

(15) γ=2−Λ

where Λ denotes the angular cost. When α→0, the distance cost decreases significantly, and when α→π/4, the distance cost is elevated.

(3) Shape Cost

Shape cost is defined by Equation (16):

(16) $$\Omega =\sum_{t=w,h}(1-e^{-\omega_{t}}{)}^{\theta }$$

where $\omega_{t}$ and θ are hyperparameters used to adjust the shape of the shape cost function. The value of θ reflects the shape cost of different datasets, when θ is set to 1, the model will optimize the aspect ratio of the detection frame and restrict its free movement. To determine the value of θ, the authors of YOLOv7 used a genetic algorithm to conduct experiments on different datasets, setting the range of θ from 2 to 6. The formulas for $\omega_{w}$ and $\omega_{h}$ are given in Equation (17) and Equation (18), respectively, when t takes the values of w and h.

(17) $$\omega_{w}=\frac{\left|w-w_{gt}\right|}{max(w,w_{gt})}$$

(18) $$\omega_{h}=\frac{\left|h-h_{gt}\right|}{max(h,h_{gt})}$$

where w and h are the width and height of the prediction frame, and $w_{gt}$ and $h_{gt}$ denote the width and height of the real frame, respectively. In summary, the final definition of the improved regression loss function is defined as in Equation (19):

(19) $$L_{SIoU}=1-I_{IoU}+\frac{\Delta +\Omega }{2}$$

The SIoU loss function expresses the complexity of the target detection task more comprehensively, reduces the probability that the value of the penalty term is 0, improves the regression accuracy and stability of the loss function, and reduces the prediction error. The optimized loss function is calculated using Equation (20):

(20) $$L=W_{SIoU}L_{SIoU}+W_{cls}L_{cls}$$

where $L_{SIoU}$ and $L_{cls}$ are the regression and categorization losses, respectively, and $W_{SIoU}$ and $W_{cls}$ are the weights of the regression and categorization losses, respectively.

## 4. Experiments and Discussions

### 4.1. Experiment Preparation

#### 4.1.1. Environment Configuration

Experiments were performed on an Intel(R) Xeon(R) Silver 4210 with a NVIDIA GeForce RTX 2080 with 10.8G of video memory. The deep-learning framework used was PyTorch (1.8.1+cu102), and the Python version was 3.7. Multi-threaded data reading was disabled due to device limitations.

#### 4.1.2. Dataset

In order to validate the detection performance of the proposed SMA-YOLO model, the experiments in this paper were evaluated using the UA-DETRAC dataset [52]. This dataset consists of more than 80,000 real road vehicle images, which are traffic vehicle images collected from roads in 24 different locations in Beijing and Tianjin. The images were taken from more than 60 videos that included four clearly labeled target objects: car, bus, van, and other. In the experiments in this study, the training set, validation set and test set of the dataset were divided in a ratio of 3:1:1.

#### 4.1.3. Evaluation Metrics

In this paper, average precision (AP), mean average precision (mAP) of all categories, number of parameters (Params), number of floating-point operations (GFLOPs), model size, and frames per second (FPS) were used as the evaluation metrics.

#### 4.1.4. Implementation Details

The input images were uniformly resized to 640 × 640 pixels during training before being fed into the target detection model. All models used in this study’s experiments employed pre-training weights obtained from training on the COCO-Train2017 dataset. This included the backbone network, MobileNetV3, which was initialized with these pre-trained weights. We fine-tuned the entire model, including the backbone and the detection head, during the training process. This approach ensured that all models benefitted from the rich feature representations learned on the COCO-Train2017 dataset.

The training phase was set to 80 epochs with a batch size of 64. The initial learning rate was 1 × 10^−3^, decaying to a final rate of 1 × 10^−5^ using cosine annealing. Momentum decay and weight decay were configured at 0.937 and 0.0005, respectively. Model parameters were optimized using the Adam optimizer. During testing, the confidence threshold was 0.1, the IoU threshold was 0.25, and each image was limited to 100 prediction boxes. For evaluation, MINOVERLAP was set to 0.5 to compute mAP_0.5_.

### 4.2. Experimental Results and Analysis

#### 4.2.1. Ablation Study

In order to verify the improvement effect of each improvement module proposed in this paper in terms of detection performance compared to the YOLOv7 model, ablation experiments were conducted, and the results are shown in Table 2.

From Table 2, it can be seen that using the YOLOv7 model as the baseline model, the addition of the SimAM attention mechanism reduced the parameters and computation of the model by 25.88% and 66.48%, respectively. The model size was reduced by 22.84%, the mAP improved by 0.47%, and the FPS improved by 42.46%. After the recalculation of the loss function using SIoU, the model’s parameters, computation and size were not significantly changed, but the mAP improved by 0.69% and the FPS decreased by 19.94%, which demonstrates a more significant improvement in the detection accuracy of the model with only a small reduction in FPS. After replacing the backbone network with MobileNetV3, although the mAP slightly decreased by 0.75%, the number of parameters of the model and the computation were reduced by 88.17% and 90.22%, respectively. The size of the model was reduced by 79.35%, and the FPS improved by 97.12%. After replacing the activation function with ACON, the number of parameters, the amount of computation, and the size of the model were improved by 1.43%, 0.47%, and 3.28%, respectively, the model’s mAP improved by 0.44%, and the FPS improved by 3.97%. Finally, by incorporating all the improved techniques into one model, the resulting SMA-YOLO model achieved a 88.20% reduction in parameters, a 90.12% reduction in computation, a 79.42% reduction in model size, a 0.28% improvement in mAP, and a 66.77% improvement in FPS when compared to the benchmark model YOLOv7.

Analyzing the data in Table 2, we observed that replacing the backbone of YOLOv7 with MobileNetV3 effectively reduced the number of model parameters and increased the FPS. Specifically, the introduction of MobileNetV3 as the backbone led to a significant reduction in computational complexity, thereby enhancing the inference speed of the model. In contrast, SMA-YOLO introduced additional modules such as SimAM, SIoU, and ACON into the YOLOv7+MobileNetV3 model. While keeping the parameters roughly the same, these enhancements increased the mAP but reduced the FPS. This trade-off is due to the additional computational overhead introduced by these advanced modules, which improve the accuracy of the model at the cost of increased computational requirements.

The experimental results show that our proposed SMA-YOLO model outperforms the initial YOLOv7 model in terms of complexity, occupied memory size, detection speed and accuracy.

#### 4.2.2. Comparison with State-of-the-Art Methods

In this section, using the same experimental conditions, the proposed SMA-YOLO model and state-of-the-art approaches were trained on the same training set and tested on the same test set, and the results are shown in Table 3.

From the experimental results shown in Table 3, it can be seen that compared with the YOLOv7 model, the proposed model SMA-YOLO’s size is 79.42% smaller, the FPS is improved by 66.77%, the number of parameters is reduced by 88.20%, the computation is reduced by 90.12%, and the mAP is improved by 0.28%. Compared to classical models preceding YOLOv7 (Table 3, rows above YOLOv7), SMA-YOLO demonstrates significantly enhanced lightweight characteristics, achieving accelerated detection speed, reduced parameter count, and lower computational complexity while maintaining superior accuracy. Compared to newer YOLO variants succeeding YOLOv7 (Table 3, rows below YOLOv7), SMA-YOLO retains detection accuracy advantages while strategically balancing speed–precision trade-offs.

#### 4.2.3. Comparison of Visualization Results

**Figure 5:**
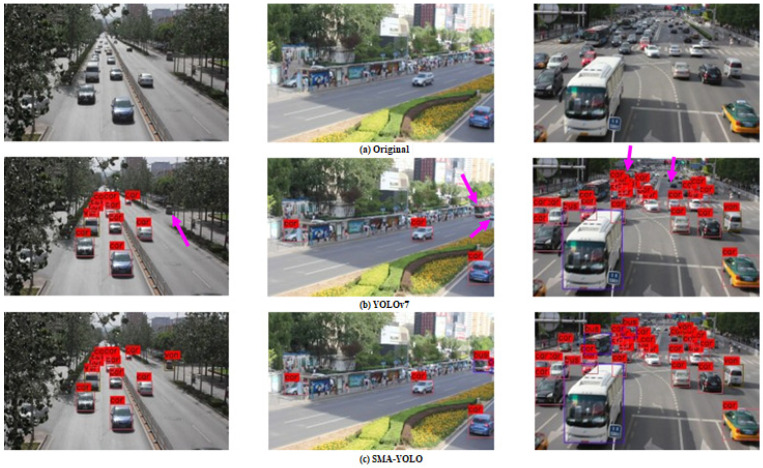
Comparison of detection results between YOLOv7 and SMA-YOLO in a road scene.

**Figure 6:**
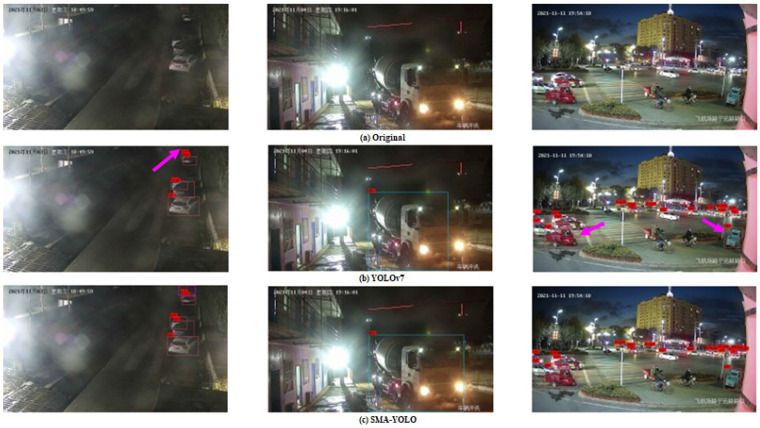
Comparison of detection results between YOLOv7 model and SMA-YOLO in a night scene.

In order to compare the detection effect of the proposed SMA-YOLO model with respect to the YOLOv7 model, we selected some representative detection results for comparison, and the results are shown in Figure 5 and Figure 6.

Comparing the three images in the first column of Figure 5, it can be seen that the proposed SMA-YOLO model was able to correctly detect the trucks that were missed by the YOLOv7 model, indicated by pink arrows. Comparing the three images in the second column of Figure 5, it can be seen that the YOLOv7 model missed the buses and the cars indicated by pink arrows in the distance, while the proposed SMA-YOLO model was able to accurately detect the locations and types of these two targets. As can be seen by comparing the three images in the third column of Figure 5, in terms of vehicle density, the YOLOv7 model missed the small targets in the distance and the partially covered vehicles indicated by pink arrows, while the proposed SMA-YOLO model was able to detect these missed vehicles correctly. Therefore, we may conclude that our proposed SMA-YOLO model can detect small and distant targets in the image more accurately, with better detection in complex road scenes.

The comparison results of the YOLOv7 model and the SMA-YOLO model proposed in this paper for the detection of vehicles at night are shown in Figure 6. Comparing the three images in the first column of Figure 6, it can be seen that the YOLOv7 model missed the detection of the truck indicated by the pink arrow due to insufficient light and strong interference at night, while the proposed SMA-YOLO model successfully detected the location of the truck and was able to identify its category. Comparing the three images in the second column of Figure 6, it can be seen that the lights of the mixer truck were highly intrusive and the far rear lighting of the truck was very harsh. Although the YOLOv7 model was able to detect the mixer truck, the location and size of the detection frame were incorrect, while the detection frame generated by the proposed SMA-YOLO model more closely matched the outline of the mixer truck. Comparing the three images in the third column of Figure 6, it can be seen that the YOLOv7 model incorrectly classified the tricycle as a car (as indicated by pink arrows), while the SMA-YOLO model was able to classify it correctly. These experiments show that the proposed SMA-YOLO model exhibited more accurate detection of ambiguous vehicles in complex nighttime scenes and generated detection frames that fit the contours of the detected vehicles more closely.

### 4.3. Discussion

In the field of object detection, there is often a trade-off between model speed (FPS) and accuracy (mAP). As shown in Table 3, SMA-YOLO demonstrates significantly enhanced lightweight characteristics compared to classical models preceding YOLOv7 (Table 3, rows above YOLOv7). SMA-YOLO achieves accelerated detection speed, reduced parameter count, and lower computational complexity, while maintaining superior accuracy. This is due to the incorporation of advanced modules such as the SimAM attention mechanism, which enhances the detection of small objects and overall accuracy but increases computational complexity.

Compared to newer YOLO variants succeeding YOLOv7 (Table 3, rows below YOLOv7), SMA-YOLO retains detection accuracy advantages while strategically balancing speed–precision trade-offs. For instance, while models like YOLOv5 and YOLOv7 demonstrate higher FPS, SMA-YOLO achieves a higher mAP, making it suitable for applications where detection accuracy is paramount. These models employ more lightweight network structures, sacrificing some detection accuracy to achieve faster inference speed.

A critical limitation involves accuracy degradation under adverse weather (e.g., heavy rain/fog), where compromised image quality impedes object detection. While current evaluations use standard datasets under typical conditions, future work will rigorously assess robustness on specialized benchmarks like the RTTS Hazy Dataset to quantify weather-induced performance drops and develop adaptive preprocessing modules for real-world deployment resilience.

The model’s efficacy diminishes with low-resolution images due to insufficient feature detail as SMA-YOLO’s design prioritizes high-resolution inputs leveraging SimAM. Future iterations will incorporate super-resolution preprocessing and lightweight architectures optimized for low-quality visuals, ensuring reliable detection in resource-constrained environments.

## 5. Conclusions

Aiming to address the problems that current vehicle detection algorithms are still not effective enough in complex traffic road scenarios and are difficult to deploy in edge devices with limited computing resources and memory, this paper has proposed SMA-YOLO, an improved vehicle detection model based on the YOLOv7 model. To enhance the flexibility and generalization performance of the vehicle detection model to meet the needs of different environments, we have made several key improvements, which include introducing the SimAM attention mechanism, adopting MobileNetV3 for feature extraction, replacing SiLU with ACON activation functions, and substituting CIoU with SIoU loss calculation. These modifications further reduced the model’s size without decreasing its accuracy and significantly improved the detection speed. Future research will focus on employing the proposed model in resource-constrained embedded devices and investigating its performance under real-world conditions, including harsh weather and low-resolution images.

## Figures and Tables

**Table 1:** Network structure of MobileNetV3-Small [16].

Input	Operator	Exp Size	#Out	SE	NL	s
224^2^ × 3	conv2d, 3 × 3	-	16	-	HS	2
112^2^ × 16	bneck, 3 × 3	16	16	√	RE	2
56^2^ × 16	bneck, 3 × 3	72	24	-	RE	2
28^2^ × 24	bneck, 3 × 3	88	24	-	RE	1
28^2^ × 24	bneck, 5 × 5	96	40	√	HS	2
14^2^ × 40	bneck, 5 × 5	240	40	√	HS	1
14^2^ × 40	bneck, 5 × 5	240	40	√	HS	1
14^2^ × 40	bneck, 5 × 5	120	48	√	HS	1
14^2^ × 48	bneck, 5 × 5	144	48	√	HS	1
14^2^ × 48	bneck, 5 × 5	288	96	√	HS	2
7^2^ × 96	bneck, 5 × 5	576	96	√	HS	1
7^2^ × 96	bneck, 5 × 5	576	96	√	HS	1
7^2^ × 96	conv2d, 1 × 1	-	576	√	HS	1
7^2^ × 576	pool, 7 × 7	-	-	-	-	1
1^2^ × 576	conv2d 1 × 1, NBN	-	1024	-	HS	1
1^2^ × 1024	conv2d 1 × 1, NBN	-	k	-	-	1

**Table 2:** Results of the ablation experiments in terms of parameters, GFLOPS, model size, mAP, and FPS.

Method	Params (10^6^)	GFLOPS (G)	Model Size (M)	mAP	FPS
YOLOv7	37.87	105.3	71.44	99.09%	10.08
YOLOv7+simAM	28.07	35.3	55.12	99.56%	14.36
YOLOv7+SIoU	37.24	105.3	71.44	99.77%	8.07
YOLOv7+MobileNet	4.48	10.3	14.75	98.35%	19.87
YOLOv7+ACON	38.41	105.8	73.78	99.53%	10.48
SMA-YOLO	4.47	10.4	14.7	99.37%	16.81

**Table 3:** Comparison results of model size, speed, accuracy, number of parameters and GFLOPs using the UA-DETRAC dataset.

Method	Input Size	Model Size (M)	FPS	Params (10^6^)	mAP	GFLOPs (G)
SSD [35]	300 × 300	94.4	10.56	24.01	76.58%	61.105
YOLOX-s [53]	640 × 640	34.7	36.7	8.96	92.55%	26.642
EfficientDet [36]	512 × 512	16.16	44.8	3.88	92.78%	4.64
Faster R-CNN (VGG16) [12]	600 × 600	521.65	27.36	136.70	94.86%	0.273
YOLOv4 [14]	416 × 416	245.01	29.24	64.02	95.65%	59.78
Faster R-CNN (Resnet50) [12]	600 × 600	108.66	27.84	28.36	97.05%	0.0566
YOLOv5 [54]	640 × 640	27.50	26.88	7.09	97.12%	16.402
Centernet [55]	512 × 512	125.25	23.63	32.72	97.64%	69.942
PP-YOLO [56]	640 × 640	203.13	11.58	4.60	98.73%	45.12
YOLOv7 [15]	640 × 640	71.44	10.08	37.87	99.09%	105.3
YOLOv8n	640 × 640	12.02	292.54	3.01	97.41%	8.10
YOLOv9s	640 × 640	28.68	105.33	7.17	98.74%	26.70
YOLOv10n	640 × 640	9.06	289.32	2.27	96.32%	6.50
SMA-YOLO (ours)	640 × 640	14.7	16.81	4.47	99.37%	10.4

## Data Availability

The UA-DETRAC dataset [52] was used to validate the models.
